# Genetic Characterization, Current Model Systems and Prognostic Stratification in PAX Fusion-Negative vs. PAX Fusion-Positive Rhabdomyosarcoma

**DOI:** 10.3390/genes12101500

**Published:** 2021-09-25

**Authors:** Carina A. Dehner, Amy E. Armstrong, Marielle Yohe, Jack F. Shern, Angela C. Hirbe

**Affiliations:** 1 Department of Pathology and Immunology, Washington University School of Medicine, St. Louis, MO 63110, USA; cdehner@wustl.edu; 2 Division of Pediatric Hematology/Oncology, Washington University, St. Louis, MO 63110, USA; armstrongae@wustl.edu; 3 Pediatric Oncology Branch, Center for Cancer Research, National Cancer Institute, Bethesda, MD 20892, USA; marielle.yohe@nih.gov (M.Y.); john.shern@nih.gov (J.F.S.); 4 Division of Oncology, Department of Medicine, Washington University School of Medicine, St. Louis, MO 63110, USA; 5 Siteman Cancer Center, Washington University School of Medicine, St. Louis, MO 63110, USA

**Keywords:** rhabdomyosarcoma, embryonal rhabdomyosarcoma, alveolar rhabdomyosarcoma, fusion-negative RMS, fusion-positive RMS

## Abstract

Rhabdomyosarcoma (RMS) is the most common soft tissue sarcoma in children and adolescents and accounts for approximately 2% of soft tissue sarcomas in adults. It is subcategorized into distinct subtypes based on histological features and fusion status (*PAX-FOXO1/VGLL2/NCOA2*). Despite advances in our understanding of the pathobiological and molecular landscape of RMS, the prognosis of these tumors has not significantly improved in recent years. Developing a better understanding of genetic abnormalities and risk stratification beyond the fusion status are crucial to developing better therapeutic strategies. Herein, we aim to highlight the genetic pathways/abnormalities involved, specifically in fusion-negative RMS, assess the currently available model systems to study RMS pathogenesis, and discuss available prognostic factors as well as their importance for risk stratification to achieve optimal therapeutic management.

## 1. Introduction

Rhabdomyosarcoma (RMS) is the most common soft tissue sarcoma in children and adolescents, with an incidence of 0.44/100,000 cases per year [1], while it is exceedingly rare in adults [2]. It has a predilection for the head and neck region [3] and is characterized by expression of myogenic markers despite failure of complete skeletal muscle differentiation [4]. While the pathogenesis of fusion-positive (FP) RMS is dominated by the generation of chimeric genes encoding for fusion proteins acting as oncoproteins that affect growth, differentiation, and proliferation, the pathobiology of fusion-negative (FN) RMS remains poorly understood. This review focuses on genetic abnormalities, known signaling pathways affected in FN-RMS, and highlights current existing model systems used to study RMS biology. Finally, we summarize the current schemes of risk stratification.

## 2. Histologic Sub-Classification

Histologically, four main subtypes are recognized in the current World Health Organization (WHO) classification [5]: embryonal (ERMS), alveolar (ARMS), spindle cell/sclerosing (SCRMS) and pleomorphic RMS (PRMS). ERMS and ARMS are the two major subtypes, while SCRMS and PRMS are significantly less common.

### 2.1. ERMS

**Figure 1:**
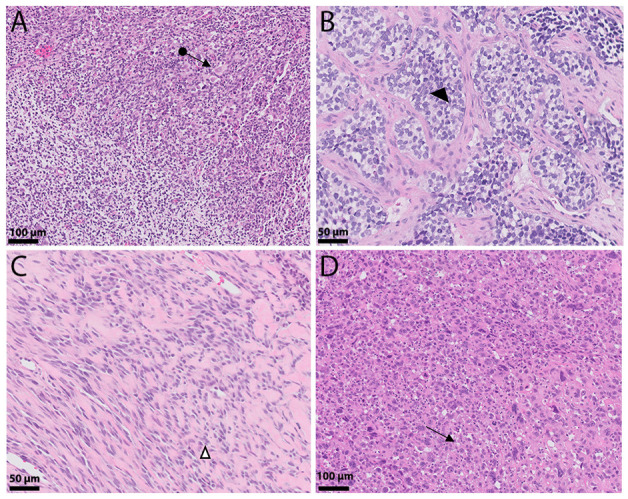
Histologic landscape of rhabdomyosarcoma. (**A**) Embryonal rhabdomyosarcoma composed of primitive round and spindled cells reminiscent of skeletal muscle cells (←●). (**B**) Alveolar rhabdomyosarcoma: Nests composed of hyperchromatic round cells intervened by fibrous septae, giving it an alveolar (◄) appearance. (**C**) Spindle/sclerosing rhabdomyosarcoma shows tumor cells arranged in cords (∆) that are set in a densely hyalinized eosinophilic background stroma. (**D**) Pleomorphic rhabdomyosarcoma presents with epithelioid tumors exhibiting significant nuclear pleomorphism with occasional cross striations and multinucleated cells (←).

Embryonal rhabdomyosarcoma is the most common subtype, with a yearly incidence of 4.6 per million children in the United States under the age of 20 [6]. Histologically, it recapitulates different stages of the development of normal skeletal muscle. Typical ERMS will frequently show alternating cellular and hypocellular, sometimes myxoid, areas with primitive spindled to ovoid cells (Figure 1A). Along with these, a second population of round cells with prominent eosinophilic cytoplasm and occasional cross-striations is notable, characteristic of differentiating rhabdomyoblasts [7]. High levels of differentiation are associated with improved overall survival compared to lower levels of differentiation [8]. The botryoid subtype contains a distinctive linear arrangement of tumor cells abutting the epithelial surface, the so-called cambium layer, with underlying sparsely cellular regions composed of small round blue cells within an edematous background. This variant commonly presents as a vaginal mass in a young female and is associated with a superior prognosis. Rarely, ERMS may show significant cytologic pleomorphism or “anaplasia”, which may be focal or diffuse and usually portrays an unfavorable outcome [9]. Focal to moderate positivity of immunohistochemical studies, MyoD1 and myogenin can help support the diagnosis.

### 2.2. ARMS

Alveolar rhabdomyosarcoma is morphologically distinct from ERMS, due to its growth pattern: tumor cells are arranged in nests that are separated by fibrous septae with loss of cellular cohesion in the center, leading to an alveolar growth pattern (Figure 1B). Solid forms may lack the alveolar pattern entirely and can, therefore, present a diagnostic challenge. Cystic areas are commonly noted, and mitotic figures are frequent. Rhabdomyoblastic differentiation may be encountered but is not always present. Bizarre and/or multinucleated giant cells are an important feature [10]. Most ARMS are driven by a fusion involving the *FOXO1* gene at 13q14.11 and either *PAX3* (2q36.1) or *PAX7* (1p36.13) [11,12,13]. Sorensen and colleagues [14] showed that these fusions are not only specific for ARMS, but they are also prognostically important, with *PAX3-FOXO1* being associated with an inferior outcome compared to *PAX7-FOXO1* [14]. Nonetheless, only 85% of ARMS show these characteristic fusion genes [15,16], with rare cases showing alternative novel fusions involving *NCOA1* or *FGFR1*, for instance [17]. This shows that, while ARMS and ERMS are easily distinguished in their classic histologic forms and traditionally were correlated with inferior and superior prognosis, respectively, it eventually became clear that these tumors can occasionally show significant overlap. Myogenin and MyoD1 are usually more diffusely positive than in ERMS.

### 2.3. SCRMS 

Only recently has there been appreciation that sclerosing and spindle cell RMS are most likely related entities. Spindle cell RMS is characterized by fascicular growth of spindle cells, resembling leiomyosarcoma. Especially in young children, the tumor may present as a deceptively bland lesion composed of low-grade appearing spindle cells recapitulating the myotubule stage of differentiation (Figure 1C). Rhabdomyoblasts may or may not be present; however, MyoD1 and myogenin are usually positive and a subset of SCRMS shows the presence of *MYOD1* (p.Leu122Arg) mutation, which occurs in its binding domain and leads to transactivation and *MYC*-like functions, leading to sustained proliferation and early inhibition of myogenic differentiation [18]. Lesions in older adults are usually more cellular and atypical [19,20,21]. Sclerosing RMS presents with prominent hyalinization/sclerosis almost resembling primitive osteoid or cartilage [19] with spindled to round tumors cells growing in cords in between. Occasionally, tumors will show a micro-alveolar growth [22,23].

### 2.4. PRMS

Lastly, pleomorphic rhabdomyosarcoma is an uncommon variant, most often presenting in adults older than the age of 45 [24,25,26,27]. This tumor is distinguished by loosely arranged, large, round or pleomorphic cells with hyperchromatic nuclei and prominently eosinophilic cytoplasm (Figure 1D). While morphologically, it may be difficult to distinguish from undifferentiated pleomorphic sarcoma, these tumors typically display strong and diffuse desmin expression with occasional co-expression of MyoD1 and myogenin. 

Several studies in the last two decades have shown the importance of fusion status, finding that FN ARMS are clinically and molecularly similar to ERMS [28,29,30,31]. The current Children’s Oncology Group (COG) clinical trial for intermediate-risk RMS (ARST1431) is using FOXO1 fusion status rather than histology for study eligibility, as it is more predictive of outcome [32,33]. This fits with the recent understanding that the absence of either of the *FOXO1* fusions is more suggestive of a diagnosis of ERMS with a primitive phenotype [10], or at least behave more like ERMS [30,34,35].

## 3. Genetics of ERMS

**Figure 2:**
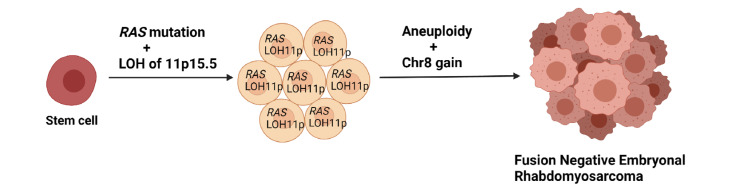
Proposed evolution of FN-RMS adapted from Chen et al. [50].

Although ERMS can be associated with syndromes, such as neurofibromatosis 1 (NF1), Costello [36], Noonan or Li-Fraumeni syndrome, or as part of the DICER1 syndrome [29], sporadic cases are much more common. Interestingly, while ARMS are most commonly near-tetraploid, 68% of ERMS exhibit hyperdiploid profiles [37]. These tumors will frequently show whole-chromosome gains, in particular, polysomy 8 [30,34,38,39,40,41], which has been seen in other cancers, such as Burkitt lymphoma [42] or solid tumors, including desmoid fibromatosis [43] and lipoblastoma [44,45], but also gains of chromosomes 2, 11, 12, 13 and/or 20 have been seen in ERMS. Additionally, FN-RMS can show loss of heterozygosity at Chr11p15.5 [46,47] (Figure 2), which has also been seen in Wilms tumor or hepatoblastoma associated with Beckwith–Wiedemann syndrome [48,49]. This locus contains imprinted genes encoding for *IGF2* and other genes, such as *CDKN1C*, with *IGF2* overexpression being an almost universal phenomenon [47]. Another fairly common copy number change is the low level amplification of *MDM2* [50].

It is known that oncogenic *RAS* is critical for FN-RMS survival, as stable knockdown of *NRAS* leads to increased apoptosis [51]. This has been corroborated by large-scale mutational analysis using whole genome sequencing on a group of ERMS, which demonstrated the vast mutational heterogeneity, including SNVs and indels in cancer genes, such as *NRAS*, *KRAS*, *TP53*, *NF1* and *HRAS* [50], with RAS pathway mutations being the most common pathogenic SNV. While mutations in any of the three isoforms of *RAS* may act as a driver mutation, the downstream consequences have not been fully elucidated [52]. Even a mutation in *NF1*, a RAS GTPase-activating protein, will lead to aberrant RAS signaling. *BRAF* and *PIK3CA* mutations have been seen as well; however, the results of these mutations in ERMS have not yet been characterized.

About 7% of FN-RMS are associated with *FGFR4* mutations, which, according to microarray studies, are differentially expressed in RMS [35,36]; mutations in the tyrosine kinase domain may result in reduced apoptosis and increased proliferation [18]. Additionally, novel mutations in F-Box and WD repeat domain-containing 7 (*FBXW7*), and *BCOR* have been identified [31].

Recent data showed that, in addition to the genomic heterogeneity, FN-RMS commonly harbor more than one single mutation, with 37% of their cohort possessing two or more mutations. This is important, as tumors with more than two mutations show significantly worse event-free survival (EFS) [53]. Most commonly, the co-occurrence of variants in tumor suppressor genes *NF1*, *TP53* and *BCOR* was identified [53], which may explain why targeting *RAS* or *PI3K* pathways alone or in combination has not resulted in sufficient cytotoxicity, as more than one pathway may have to be targeted [54]. *TP53* mutations specifically, while present in about 13% of FN-RMS, have been shown to be associated with worse EFS [53]. Mutations in *MYOD1*, found predominantly in FN-RMS with spindle or sclerosing features, and also in rare cases of ERMS and RMS NOS, are associated with rapid progression and overall dismal outcome. *MYOD1* mutations are not mutually exclusive with *RAS*, and also have frequently occurred in combination with deletions in *CDKN2A*, which in itself is correlated with worse EFS [53].

## 4. Epigenetics

It is well established that FP and FN-RMS subtypes portray different methylation patterns, with FP-RMS showing increased methylation of genes targeted by the polycomb repressive complexes 1 and 2 (PRC1 and PRC2) [55]. While PRC2 tri-methylates lysine-27 of histone H3, its product H3K27me3 recruits PRC1, the main transcription silencer of Polycomb group proteins (PcGs) [56]. Activity of PcGs is important for regulation of the transcriptome during embryogenesis [57].

Interestingly, FN-RMS tumors are clustered more with normal muscle than FP-RMS, indicating that FN-RMS are epigenetically “closer” to a “normal” state [55]. Further, a panel of 11 genes (CpG sites) is enough to confidently separate FP- from FN-RMS [58]. While RAS mutant and wild-type FN-RMS exhibit different methylation profiles, those with wild-type *RAS* are the tumors more closely resembling “normal skeletal muscle”. This is interesting given that wild-type FN-RMS still harbor mutations in RAS pathway genes, including *NF1* and *SOS1* [58].

Another, very recently discovered epigenetic mechanism involving oncogenic *RAS*, through MAPK signaling, drives FN-RMS proliferation and, subsequently, suppresses expression of *MYOG*, which prevents myogenic differentiation via induction of H3K27 acetylation, chromatin opening and gene transcription [51]. Additionally, work from Stewart and colleagues showed the importance of epigenetic changes, including DNA methylation, protein and histone modifications, comparing ERMS and ARMS epigenetically, leading to the conclusion that ARMS tumors are arrested at a later stage of muscle development than ERMS [59]. A further interesting finding was that 7.4% of FN-RMS are affected by mutations in the transcriptional repressor *BCOR*, commonly mutated in other tumors, including retinoblastoma [60] and medulloblastoma [61], which seems to interact with histone deacetylases [31].

## 5. Signaling Pathways in RMS

In many ways, RMS resembles embryonic skeletal muscle, and a failure of complete differentiation is the proposed mechanism of disease. Importantly, RMS can occur at sites that lack skeletal muscle, such as the salivary gland, gallbladder, and bladder, which places a true skeletal, muscle-derived origin into question [62].

The Sonic hedgehog (SHH) pathway is critical in early stages of skeletal muscle development, and many studies have shown its importance in ERMS pathogenesis. Its key players are HH ligands, the transmembrane proteins patch (PTCH1), smoothen (SMO) and the transcription factor GLI. Devoid of HH ligand, PTCH1 represses SMO. If HH ligands bind to PTCH1, SMO is released and further activates GLI1. Several FN-RMS models have shown to be associated with aberrant SHH signaling [63,64,65]. Additionally, we know that a third of ERMS have loss of Chr9q22, which contains the *PTCH1* gene [39], and over half of ERMS have gain of Chr12q13.3, which contains *GLI1*. Nonetheless, no activating mutations have been described, and thus, the precise role of SHH pathway activation remains unclear [66]; however, we do know that inhibition of HH signaling regulates MyoD transcriptional activity during myogenesis [67]. However, alterations in this pathway do correlate with poor outcome in FN-RMS [68]. Further, SHH seems to control self-renewal of FN-RMS; its inhibition has been shown to reduce chemotherapy resistance [69]. In addition, there is new evidence that oncogenic RAS mutations (*HRAS*, *KRAS* and *NRAS*) inhibit GLI1 via the MEK/ERK pathway but concurrently lead to increased proliferation and oncogenicity [70].

Another important gene in embryonic and postnatal skeletal myogenesis is *NOTCH1* [71,72]. Studies have shown that *HEY1*-*NOTCH* signaling is upregulated in ERMS cell lines and tumor samples, compared to normal skeletal muscle. Further, both genetic and pharmacologic inhibition of Notch signaling is able to block ERMS tumorigenesis both in vitro and in vivo [73]. One possible mechanism is knocking down downstream target *HEY1*, whose protein product usually associates with promotor regions of two important myogenic genes, *myogenin* and *Mef2C*, induces expression of pro-myogenic skeletal muscle transcription factors and possibly induces multinucleated myotube formation. Belyea and colleagues [73], however, found that *HEY1* knock-down was not sufficient to induce terminal differentiation.

The third important player in embryonic signaling pathways affecting skeletal muscle development is *WNT*. Side by side with Notch and Hedgehog signaling, *WNT* is important for the regulation of the progression of muscle stem cells toward lineage-committed progenitors. While MYOD is activated independently of β-catenin [74], MYF5 activation does depend on β-catenin [75]; both are important in early myogenesis and are expressed mutually exclusively, each being important for sustained cell proliferation [76]. Β-catenin is also required for dermomyotome and myotome formation [77]. However, its role in rhabdomyosarcomagenesis is superficially understood. We do know that a subgroup of *PAX* gene fusion-negative RMS shows activating point mutations in *CTNNB1* on Chr3 [47]. A *p^−/−^/c-fos^−/−^* ERMS mouse model showed that, while Wnt2 was overexpressed in ERMS compared to normal muscle myoblasts, Wnt/β-catenin signaling was downregulated; reactivation of this pathway could induce MyoD expression, thereby promoting terminal differentiation [78]. This finding suggests that therapeutic activation of the Wnt pathway could represent a potential treatment approach.

**Figure 3:**
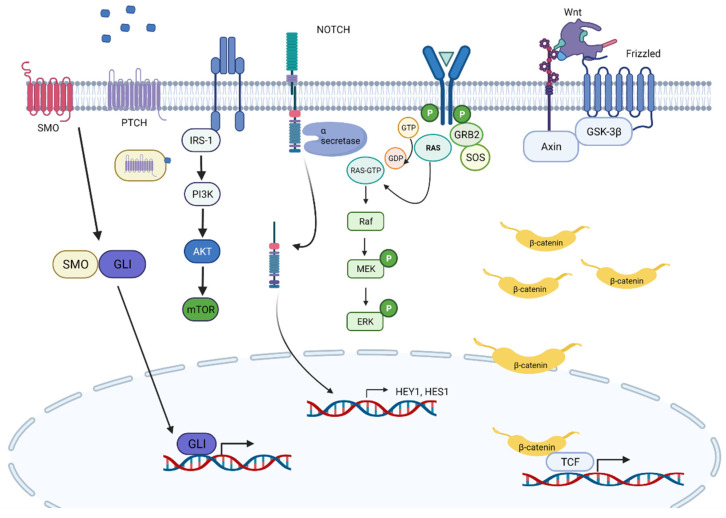
Overview of important signaling pathways in FN-RMS pathogenesis.

Furthermore, the Hippo pathway transducer YAP1 has been shown to be elevated in ERMS. Via its interaction with TEAD1, it is able to upregulate pro-growth and oncogenic genes. In addition, YAP1-TEAD1 contributes to the differentiation block by interfering with MYOD1 and MEF2, which can be reversed by normalizing YAP1 expression; differentiation may be achieved, making YAP1 an important therapeutic target [79]. Slemmons and colleagues took this a step further and demonstrated that inhibition of YAP1 in a Hippo-dependent manner (through RASSF alterations) can be achieved by treatment with a DNA methyltransferase inhibitor (DNMTi) or in a Hippo-independent manner (through YES1) using dasatinib, a Src family kinase inhibitor. Combined treatment with DNMTi and a Src inhibitor was able to reduce cell growth and induce apoptosis, suggesting this as a novel therapeutic strategy, mostly for refractory or recurrent RMS, particularly for ARMS [80] (Figure 3).

More recently, the MAPK pathway in RAS-driven FN-RMS was shown to promote RMS pathogenesis while inhibiting MYOG expression. This effect can be reversed by inhibiting MEK, using the MEK inhibitor trametinib, which subsequently leads to differentiation of *RAS*-mutated FN-RMS. This finding was further supported, using xenograft models in which trametinib was able to inhibit tumor growth and induce skeletal muscle differentiation. Importantly, after the addition of IGF1R inhibitor BMS-754807, which has been shown to work as combination therapy in a variety of *RAS*-driven cancers, such as acute leukemia, non-small cell lung cancer and colorectal carcinoma [81,82,83], tumor regression was achieved, which represents a promising therapeutic opportunity [51].

The mTOR pathway seems to contribute to RMS invasion, while inhibition of it seems to diminish cellular migration, invasion and angiogenesis in RMS models. Specifically, mTOR is activated downstream of AKT, which then leads to inactivation of 4E-BP1 (a eukaryotic initiation factor) and activation of S6K1. The combination of these events leads to HIF1α expression, a transcription factor for anti-hypoxic gene expression [84]. There is recent evidence that targeting both the PI3K/mTOR and the MEK/ERK pathways can synergistically inhibit RMS cell growth in vitro and in vivo [85,86]. Additionally, temsirolimus (an mTOR inhibitor which is known to be converted to rapamycin in vivo) showed clinical activity in heavily pre-treated patients with RMS in a COG randomized phase 2 study (ARST0921) [87] and is currently being evaluated in a large phase 3 study for upfront treatment of intermediate-risk RMS patients.

Within the last few decades, significant progress has been made regarding the understanding of myogenesis and, thus, what may underlie the pathogenesis of RMS. Additionally, with the development of model systems, such as induced pluripotent stem cells (iPSCs) or patient-derived xenografts (PDX), there is excitement for translational researchers, as these novel model systems could be utilized to study RMS pathobiology and to identify new targets.

## 6. Rhabdomyosarcoma Cell Lines

To date, there are 30 commonly used RMS cell lines: 18 are embryonal and 12 are derived from tumors with alveolar histology. Hinson et al. [88] nicely summarized all 30 available RMS cell lines with the goal of aiding scientists in choosing the most suitable line to investigate their hypothesis. Some lines have caveats, such as prior treatment, or possible incongruity between the original tumor and the resulting cell line. Importantly, some of the cell lines are derived from the same parental tumor, as is the case, for instance, for RH36, Birch, RH30 and RMS13 [88], which may not be immediately recognized, due to different terminology. Unfortunately, despite the quantity of available cell lines, there is a significant lack of lines derived from untreated tumors, as most of the lines are developed from treated tumors or distant metastases. For example, JR1 cells stem from a lung metastasis of a 7-year-old female patient. This is important to consider, as cells arising from relapses or metastases may have acquired additional/different genetic changes. Therefore, it is crucial to seek additional model systems to better study RMS pathobiology.

Linardic et al. [89] used a different approach by transforming both human fetal skeletal muscle cell (SkMC) precursors and postnatal human skeletal muscle myoblasts (HSMM) so that these would express SV40 large and small T antigen oncoproteins (T/t-Ag), human telomerase catalytic subunit (hTERT) and oncogenic HRAS-G12V, with the aim of recapitulating a human rhabdomyosarcoma-like tumor model that would allow for studying the underlying disease mechanisms. Interestingly, the HSMM-derived xenografts resembled histologically ERMS, while SkMCs did not show any morphological features reminiscent of ARMS or ERMS by giving rise to vastly heterogenous sarcoma histology. This suggests that implementation of identical molecular changes in two different cell model systems lead to different tumor morphologies, which demonstrates that cell of origin is important for rhabdomyosarcoma histology.

## 7. Mouse Models

Despite its fairly low frequency compared to *RAS*, *TP53* mutation represents a significant risk for the development of ERMS, which can be used scientifically, as *Tp53* null mice will exhibit ERMS tumorigenesis (in low levels) [90] (Table 1). Another targeted pathway is Hgf/c-MET. Increased Hgf expression was shown to induce RMS tumors in transgenic mice [91]. Additionally, aberrant c-MET signaling with simultaneous INK4a/ARF inactivation are essential for rhabdomyosarcomagenesis [92]. Models affecting the SHH pathway, in particular *Ptch1*^+/−^ mice, exist, but have been associated with multi-organ tumorigenesis rather than specifically RMS formation, due to the additional development of features consistent with Gorlin syndrome [63]. *Ptch1*^+/−^ mice generally display significantly fewer aggressive RMS features, due to the greater degree of differentiation [93]. Mice lacking *Sufu* (Suppressor of fused) have been generated. However, these mice only showed tumor development (medulloblastoma and rhabdomyosarcoma) if *Tp53* was also lost [94].

A recent study investigated the utility of patient-derived xenografts (PDXs) and compared these to RMS cell lines, cell line-derived xenografts (CDXs) and parental tumors [58], looking specifically at the relationship between DNA methylation and mutational changes. Their data showed that RMS PDXs have a very similar DNA methylation pattern, compared to their parental tumors, whereas RMS cell lines and CDXs have a distinctly different methylation pattern, a finding that has been well established in other tumors, such as osteosarcoma or colon cancer [103]. For RMS, however, the use of PDX models has been limited and can be technically challenging. Usually, tumor fragments are implanted heterotopically (subcutaneously or into the renal sinus, unrelated to the site of the original tumor) or orthotopically (original site of the tumor) into an immunodeficient mouse [104]. At the same time, the preserved tumor heterogeneity makes them an invaluable resource; it is anticipated that more and more scientists will use them as a model system [105,106,107].

One possible future model may be the use of iPSCs with the aim of differentiating them into rhabdomyoblasts, as it has been done for plexiform neurofibromas and MPNST [108] as well as other cancers [108,109]. More recently, several studies reported the successful use of iPSCs in disease models of muscular dystrophy [110,111], or to study normal skeletal muscle development [112]. Even single studies have explored ARMS, using immortalized human myoblasts [113]. Thus, this model may be exploited to study FN-RMS pathobiology in the near future.

## 8. Risk Stratification

While several scientific advances within the last years have been made, the overall survival rates for pediatric patients, especially with high-risk or relapsed RMS, have not improved since the 1980s [114]. Evaluation of non-metastatic RMS patients treated on the third and fourth Intergroup Rhabdomyosarcoma Studies (IRS-III and IRS-IV) identified the prognostic significance of the histology, stage, clinical group (the amount of residual tumor after initial surgery or biopsy before the start of systemic chemotherapy) and primary tumor site. This was followed by a model in which patients were divided into treatment groups: two of low-risk, one of intermediate-risk and one of high-risk treatmentgroups [115]. When evaluating tumor sites, favorable locations were found to include the non-parameningeal head/neck, orbit, non-bladder/prostate genitourinary tract, biliary and liver; all other sites are considered unfavorable [116,117,118,119].

Furthermore, Oberlin and colleagues [120] analyzed 778 patients with metastatic RMS and established several factors correlating with worse EFS: age < 1 year or >10 years, unfavorable site of tumor, presence of three or more sites of metastatic disease and presence of bone or bone marrow involvement. After metastatic status, it is now determined that the *FOXO1* fusion status is the most important prognostic factor; it improves the risk stratification of patients with localized RMS [121,122]. Indeed, several groups demonstrated that the key factor for progression and outcome of RMS is driven by the presence or absence of the *PAX/FOXO1* fusion status [29,30].

Current risk stratification used in the United States by the COG to dictate treatment intensity integrates clinical group, age, and stage, the latter incorporating the primary tumor site, tumor size, and nodal status; the presence of metastases automatically renders a patient as Stage 4, Group IV. While histology has historically been used in risk classification schemas, fusion status is now taking precedent in the COG risk stratification system.We previously determined adequate classification of Subset 1 low-risk patients on ARST0331, given excellent 3-year failure free survival (FFS) of 88% and overall survival (OS) of 98% following administration of 22 weeks of chemotherapy, including a low cumulative cyclophosphamide dose [123]. Subset 1 included Stages 1 and 2, Group I/II or Stage 1, Group III (orbit) ERMS. In comparison, Subset 2, which included Stage 1, Group III non-orbit or Stage 3, Group I/II ERMS, had a suboptimal 3-year FFS of 70%, with a 3-year OS of 92%. Of note, ARMS was not treated on the low-risk study ARST0331, but FN-ARMS will now be treated using the same low-risk approach on ARST1431 for Stage 1, Group I/II, Stage 1, Group III (orbit) or Stage 2, Group I/II (i.e., Subset 1 per ARST0331). Additionally, Subset 2 from ARST0331 has been reclassified as intermediate-risk and is now included in the ARST1431 open study. The high-risk rhabdomyosarcoma group includes fusion-positive metastatic patients regardless of age, as well as metastatic fusion-negative or ERMS patients who are 10 years of age or older. It should be noted that there is a lack of international consensus on risk stratification in rhabdomyosarcoma [124].

Additionally, there are histologic features, beyond alveolar vs. embryonal, that have been assessed for their prognostic significance, in particular, the presence of anaplasia. Anaplasia is a histologic phenomenon that is associated with worse outcomes in other childhood cancers, such as Wilms tumor [125], and has been appreciated in cases of ERMS more commonly than in any other subtype of RMS [9]. Recent data suggest that anaplasia in RMS is not an independent adverse prognostic factor, with the caveat that larger studies may be necessary to confirm this finding [126]. However, the authors found that *TP53* mutation is associated with worse outcome, and some data have suggested a correlation between the presence of anaplasia and *TP53* mutational status [127].

## 9. Conclusions

Overall, there has been significant progress in the establishment of relevant prognostic factors; with the shift toward molecular identification of the majority of tumors, it is expected to see additional factors and increased importance of genetic stratification. The increase in new model systems has led to significant scientific progress and understanding of fusion-driven rhabdomyosarcoma; however, fusion-negative rhabdomyosarcoma pathogenesis is more heterogeneous, only partially understood, and should be an important focus. Prospective clinical trials with correlative tumor samples and uniform molecular profiling are needed to better understand which factors are associated with worse outcome and how to use the available tools to appropriately stratify these tumors. Which mutations are associated with refractory or recurrent disease and at what stage of the tumor development can we assess for these? Additionally, evaluation of the copy number variations (CNVs) has been largely unaddressed in ERMS but may offer an opportunity to uncover dysregulated molecular targets and pathways driving tumor development, progression or relapse. Large-scale analyses of CNV data in the context of sufficiently long outcome data are needed to determine their relevance in therapeutic planning for RMS. Lastly, further efforts should be made to incorporate newer subtypes into upcoming risk-classification schemas, such as tumors with *MYOD1* mutations, novel fusions or *TP*53 mutations. 

## Figures and Tables

**Table 1:** Currently existing mouse models to study ERMS [95].

Genes/Targeted Pathways	Genetic Modification in Mouse	FN-RMS	References
*RAS*	Expression of *KRAS G12D* expression (±background of heterozygous or homozygous p53)	UPS with myogenic features	[96]
*FGFR4*	Murine myoblasts expressing FGFR4^V550E^	RMS	[97]
*P53*	Knock-out of *p53* in C57BL/6	RMS	[90]
	*Trp53/FOS* double knock-out in 129Sv X C57BL/6	ERMS	[98]
	Constitutively expressed HER2/neu in Balb/c with *p53^+^*^/−^ via MMTV promotor	ERMS	[99]
	*KRAS G12V* conditional expression in adult Balb/c, expressed from *Rag2* promotor	ERMS	[100]
	Transgenic *KRASG12V* expression with concurrent knock-out of p53/gain of p53^R172H^ mutant in C57B16J/S129 mice	PRMS	[101]
*HGF*	*HGF/SF* Overexpression of HGF in albino FVB/N mice	PRMS with lung metastases	[91,92]
Sonic hedgehog	Inactivation of *Ptch* mutations	RMS	[102]
